# Phosphorylation of tumour suppressor Amotl2 by IKBKE kinase promotes YAP1 signalling, enhancing glioblastoma growth

**DOI:** 10.3389/fonc.2026.1823895

**Published:** 2026-07-24

**Authors:** Gaochao Guo, Yan Sun, Rujun Hong, Yalin Lu, Xingjie Chen, Liming Zhao, Chaoyue Li, Yang Liu, Qiang Huang

**Affiliations:** 1 Department of Neurosurgery, Henan Provincial People,s Hospital; Zhengzhou University People,s Hospital; Cerebrovascular Disease Hospital, Zhengzhou, Henan, China; 2 Tianjin Neurological Institute, Key Laboratory of Post-Neuroinjury Neuro-repair and Regeneration in Central Nervous System, Ministry of Education and Tianjin City, Tianjin Medical University General Hospital, Tianjin, China; 3 Department of Neurosurgery, Yantai Yuhuangding Hospital, Yantai, Shandong, China

**Keywords:** Amot2, glioblastoma (GBM), IKBKE, phosphorylate, Yap1

## Abstract

Inhibitor of nuclear factor kappa-B kinase subunit epsilon (IKBKE), a member of the serine/threonine kinase family, is an important oncogene in glioblastoma. IKBKE is involved in the progression of multiple tumours in glioblastoma (GBM), including tumour invasion, migration, and proliferation. Here, we report an IKBKE–angiomotin-like protein 2 (Amotl2)–yes-associated protein 1 (YAP1) axis in which IKBKE inhibits the protein expression of Amotl2, while Amotl2 regulates the nuclear transport of the YAP1 protein, which has been implicated in brain tumour development and progression. Data analysis of multiple IKBKE mRNA databases of glioma and immunohistochemical analysis of a tissue chip indicated that higher IKBKE expression was associated with higher malignancy and shorter survival in glioma patients. IKBKE downregulation significantly inhibited GBM cell proliferation and restrained tumour growth in a GBM mouse model. Moreover, IKBKE phosphorylates Amotl2, promoting Amotl2 ubiquitination and degradation, leading to YAP1 entry into the nucleus and the activation of downstream genes. In summary, our results are the first to show that IKBKE phosphorylates Amotl2 and that GBM cell proliferation is regulated by the IKBKE↑–Amotl2↓–YAP1↑ axis.

## 1 Introduction

Glioblastoma (GBM) is one of the most common malignant brain tumours, with a median survival of less than 15 months after diagnosis (1–3). This poor prognosis is due to the intrinsic ability to acquire therapeutic resistance and a high rate of recurrence (4, 5). Although treatments for GBM have improved over recent decades, the prognosis of GBM remains undesirable (6). Thus, it is important to elucidate the molecular pathogenesis of GBM to discover novel treatments.

Inhibitor of nuclear factor kappa-B kinase subunit epsilon (IKBKE) is a serine/threonine kinase (7, 8). IKBKE has been reported to be an oncogenic protein in a variety of tumours, and can regulate cell growth, migration, apoptosis, and autophagy by stimulating the NF-kb, AKT/mTOR, STAT, IRF3/7,and Hippo signalling pathways (8–15). In GBM progression, IKBKE regulates epithelial–mesenchymal transition (EMT), cell proliferation, and chemoresistance (12, 16–21). The downregulation of IKBKE expression by siRNA significantly inhibits GBM cell proliferation and invasion (22). However, the overexpression of IKBKE in gliomas can contribute to glioma cell resistance to apoptosis by activating NF-kb (18). Our previous study have reported that Amlexanox, a selective inhibitor of IKBKE, significantly inhibits proliferation and invasion and induces apoptosis in GBM cells (12, 17). Thus, IKBKE may be an important therapeutic target. However, the molecular mechanisms by which IKBKE regulates GBM progression have not been well defined.

The angiomotin (Amot) family comprises three members: Amot (p80 and p130 isoforms), Amot-like protein 1 (Amotl1), and Amot-like protein 2 (Amotl2) (23, 24). In most cancers, Amot proteins promote the proliferation and invasion of cancer cells; however, Amot proteins inhibit the growth of glioblastoma cells, ovarian cancer cells, and lung cancer cells.

Amot regulates the MAPK/ERK, Wnt/β-catenin, AMPK, and mTOR signalling pathways (24–28). Amotl2 is a membrane-associated scaffold protein that is a key regulator of the Hippo signalling pathway (29). Amotl2 has been reported to regulate yes-associated protein 1 (YAP1) through protein–protein interactions and sequester YAP1 in the cytoplasm independent of the Hippo signalling pathway (28, 29). Amotl2 also activates large tumour suppressor (LATS) kinases, which suppress YAP1 by phosphorylation, to participate in the Hippo signalling pathway (28, 29). These results identify Amotl2 as a significant regulator of the Hippo signalling pathway.

Here, we aim to clarify that IKBKE can promote the glioblastoma growth through the IKBKE–Amotl2–YAP1 axis. In this study, we found that IKBKE can phosphorylate Amotl2 at serine 166 (s166), and negatively regulate its expression, and regulate its translocation from the cytoplasm to the nucleus, leading to enhanced YAP function, which can verify that IKBKE is identified as a promising therapeutic target for glioma treatment.

## 2 Materials and methods

### 2.1 Online databases

We used mRNA expression microarray data for the non-tumour tissues and all tumour grades from the Chinese Glioma Genome Atlas (CGGA) database (http://www.cgga.org.cn). The Cancer Genome Atlas (TCGA) (http://cancergenome.nih.gov) and GSE16011 dataset (http://www.ncbi.nlm.nih.gov/geo/query/acc.cgi?acc=GSE16011) were used to validate mRNA expression levels as the validation platforms. The mRNA expression profiles of IKBKE and related prognoses of patients were also analysed.

### 2.2 Cells and tissue samples

Human GBM cell lines (U87MG) and HEK293 were obtained from the Laboratory of Neuro-Oncology at Tianjin Neurological Institute, China, and GBM cell lines were cultured in standard DMEM with 10% FBS at 37 °C, 5% CO_2_. The primary cell line G4 was derived from a GBM patient with recurrent lesions undergoing surgery at the Tianjin Medical University General Hospital, according to the protocol reported by Dong et al., and cultured in Dulbecco’s modified Eagle’s medium (DMEM)/F12 (Gibco, Billings, MT, USA) supplemented with 10% foetal bovine serum (Gibco) at 37 °C, 5% CO_2_ (30). For cell viability detection, defined cell numbers were seeded into 96-well plates with 6 technical duplicates per condition, and 3 independent biological replicates were conducted. We also clearly stated the seeding timeline and all detection time points (0, 24, 48, 72 h) for cell growth monitoring. Glioma specimens with complete clinical data were collected from Tianjin Medical University General Hospital, according to the ethical and legal standards of Tianjin Medical University General Hospital (31). Classification of CNS tumours and tumour grading were performed in compliance with the World Health Organization (WHO) criteria. None of the patients had received preoperative chemotherapy or radiation therapy. The 145 glioma specimens cover all WHO classification grades: WHO Grade I: 4 cases. WHO Grade II: 25 cases, WHO Grade III: 35 cases, WHO Grade IV (glioblastoma): 81 cases. We adopted the widely accepted H-score (histochemical score) system for semi-quantitative evaluation of IKBKE staining: a. Staining intensity: 0 (no staining), 1 (weak), 2 (moderate), 3 (strong); b. Positive cell proportion: 0–100%; c. H-score = staining intensity × percentage of positive cells, ranging from 0 to 300. Samples with H-score median cut-off were divided into IKBKE low-expression and high-expression subgroups for subsequent statistical analysis. Two independent pathologists blinded to clinical information separately scored all TMA slices to avoid subjective bias. We calculated the inter-rater reliability via Cohen’s Kappa test; the Kappa value = 0.86, indicating excellent consistency between the two raters. Any discrepant scoring results were re-reviewed under a double-headed microscope to reach a consensus score.

### 2.3 Cell proliferation assay

Cell proliferation was performed using the Cell Counting Kit-8(CCK-8); Dojindo, Kumamoto, Japan) and the EdU DNA Proliferation in Detection Kit (RiboBio, Guangzhou, China). First, transfected GBM cells were plated in 96-well plates at a density of 2.0 × 10^3^ cells/well. At the indicated time points, 10 μL of CCK-8 was added to the cells and incubated for 1.5 h. Then, the absorbance was measured at 450 nm (OD_450_) using a microplate reader (Synergy 2; BioTek, Winooski, VT, USA).

Furthermore, U87MG or G4 cells were incubated with 50 μM EdU for 2 h. Following fixation with 4% paraformaldehyde and permeabilisation with 0.5% Triton X-100, the cells were subjected to EdU staining using Apollo solution. Cell nuclei were stained with Hoechst 33342. EdU-positive nuclei were visualised under a fluorescence microscope (Olympus, Tokyo, Japan).

### 2.4 Cell colony formation assay

Transfected GBM cells were placed in 6-well plates at a density of 200 cells/well. The cells were grown for two weeks to form visible cell colonies, which were then fixed with methanol and stained with crystal violet. Colonies (≥50 cells) were counted using an inverted microscope (Olympus).

### 2.5 Construction of the lentiviral vector system and cell transfection

The target sequences of the IKBKE shRNA and the negative control-shRNA sequence were 5′-GCATCATCGAACGGCTAAATA-3′ and 5′-TTCTCCGAACGTGTCACGTTTC-3′, respectively. The lentiviral constructs were designed and provided by GeneChem (Shanghai, China). The Amotl2 siRNA target sequences used here are as follows:

si-Amotl2–1 target sequence: GGACUGGUUCACUAAUCAAdTdT; si-Amotl2–2 target sequence: GGUUCAUGUGCAUUGUUUAdTdT; si-Amotl2–3 target sequence: GAGAUGUCUUGUUAGCAUAdTdT; si-NC target sequence: UUCUCCGAACGUGUC ACGUdTdT. The siRNA kit and sh-Amotl2 lentiviral constructs were designed and purchased from HANBIO (Shanghai, China). The constructed lentiviral vectors were transfected into U87MG and G4 cells, according to the manufacturer’s protocol.

### 2.6 Plasmid construction

The IKBKE and control vector plasmids were purchased from Addgene (Watertown, MA, USA). We created IKBKE plasmid and control plasmid overexpressing lentiviral cells using GeneChem Biotechnology (Shanghai, China). The Amotl2 and control vector plasmids were designed and purchased from HANBIO. The plasmid vectors were transfected into U87MG and G4 cells, according to the manufacturer’s protocol.

### 2.7 Cytoplasmic and nuclear fractionation

Cytoplasmic and nuclear extracts were separated using a Nuclear and Cytoplasmic Protein Extraction Kit from Beyotime (Guangzhou, China) according to the manufacturer’s instructions.

### 2.8 *In vitro* kinase assay

IKBKE *in vitro* kinase assay was performed as previously described (32).

### 2.9 Mass spectrometry analysis

Mass spectrometry was used to map the phosphorylation sites of Amtol2 by IKBKE. U87MG sh-NC and U87MG sh-IKBKE cells were used for Mass spectrometry. Cell sample processing and post-stage data analysis were performed by Putian Biotechnology Co., LTD. (Tianjin, China).

### 2.10 Western blot analysis, co-immunoprecipitation analysis, and ubiquitination assay

Western blot analysis was performed as previously described (18). Antibodies against IKBKE, Amotl2, YAP1, CTGF, Cyr61, ubiquitin, and labelled antibodies (Myc and Flag) were purchased from Cell Signalling Technology (Danvers, MA, USA). P-Ser antibody was purchased from Santa Cruz Biotechnology (Dallas, Texas, USA). For co-immunoprecipitation, cells were lysed in IP lysis/wash buffer (Thermo Fisher Scientific, Waltham, MA, USA). The total cell lysates were incubated with protein G agarose (Santa Cruz Biotechnology) and mixed with anti-IKBKE or anti-Amtol2 antibody overnight at 4 °C. Immunoprecipitated proteins were resolved by SDS-PAGE. For the endogenous Amtol2 ubiquitination assay (12), cell lysates were incubated with anti-Amtol2 antibody and analysed by western blotting using anti-ubiquitin and anti-Amtol2 antibodies. For the *in vivo* ubiquitination assay (33), 293T cells were transfected with either vector (control), Flag-IKBKE, Myc-Amtol2, or HA-ubiquitin plasmids as indicated. Quantitative gray-scale analysis of total Amotl2 western blot bands was provided in **Supplementary Figure 1**.

Antibodies against the following were used in the experiments:

IKBKE (WB: 1:1000, IP: 1:100, Cell Signaling Technology, 2905), GAPDH (WB:1:4000, Utibody, UM4002), Amotl2 (WB:1:1000, IP:1:100, IHC:1:250, IF:1:200, Cell Signaling Technology, 30020), p-ser (WB:1:1000, IP:1:100, Santa Cruz Biotechnology, sc-81515), YAP1 (WB:1:1000, IHC:1:200, IF:1:100, Cell Signaling Technology, 14074),p-YAP1(s127)(WB:1:1000, Cell Signaling Technology,13008) and Myc(WB:1:1000,IP:1:250, Cell Signaling Technology,2276) and Flag (WB:1:1000,IP:1:500,Cell Signaling Technology, 1473),Ki67 (IHC:1:200,12202),CTGF (IHC:1:800,Cell Signaling Technology,3418), CYR61 (IHC:1:50,Cell Signaling Technology,39382),Ubiquitin (WB: 1:1000,Cell Signaling Technology, 3936).

### 2.11 Immunofluorescence staining

For immunofluorescence analysis, U87 and G4 cells transfected with lentiviral vectors were grown on cover slips, fixed with 4% paraformaldehyde, and incubated overnight with IKBKE or Amtol2 antibodies. The distributions of IKBKE and Amtol2 in the cytoplasm and nucleus were visualised using a confocal microscope (Olympus).

### 2.12 RNA extraction and quantitative real-time PCR

Total RNA was extracted using TRIzol reagent (Invitrogen, Waltham, MA, USA). Reverse transcription was performed using a GoScript™ Reverse Transcriptase Kit (Promega, USA). The sequences of the PCR primers (forward and reverse) were 5′-GGAGCGAGATCCCTCCAAAA-3′ and 5′-GGCTGTTGTCATACTTCTCATGG-3′ for GAPDH; 5′-GAGAAGTTCGTCTCGGTCTATGG-3′ and 5′-TGCATGGTACAAGGTCAC TCC-3′; IKBKE, 5′-gTCCCgTCgATgggTTTAgg-3′ and 5′-gCTTCTTTggCTTgCACACA-3′ for Amotl2.

### 2.13 *In vivo* tumorigenesis assay

Female BALB/C nude mice (4–5 weeks old) were purchased from the Animal Centre of the Cancer Institute at the Chinese Academy of Medical Science (Beijing, China) and used for xenograft studies. The details about the orthotopic xenograft model: (a) n=6 mice per group; (b) Mice were randomized via random number table after one-week acclimatization, with balanced baseline body weight; (c) Blinded assessment was applied for survival recording, tumor imaging and histological quantification; researchers were unaware of group assignments during data analysis; (d) 5 × 10^4^ viable GBM cells suspended in 2 μL PBS were injected per mouse; (e) Stereotactic coordinates: AP + 0.5 mm, ML ±1.8 mm, DV −2.5 mm relative to bregma; (f) Humane endpoints: &gt;20% weight loss, severe neurological dysfunction, inability to feed/drink; all animals were sacrificed at day 60 post injection. All animal procedures follow ARRIVE guidelines and were approved by institutional IACUC.

To establish the intracranial model, 5 × 10^4^ cells (U87-sh-Ctrl or U87-shIKBKE) with luciferase-encoding lentivirus were stereotaxically injected into the mice (six mice per group). Intracranial tumour growth was monitored by bioluminescence imaging at 7, 14, and 21 d using the IVIS Spectrum Live Imaging System (PerkinElmer, Branford, CT, USA). Animal research was approved by the Ethics Committee for Animal Experimentation of Tianjin Medical University General Hospital.

### 2.14 Statistical analysis

Statistical analyses were performed with GraphPad software (version8.0). A t-test was used to analyze the two-group comparison and One-way ANOVA was used to test for differences among at least 3 groups, and a least significant difference *post hoc* test was used to obtain individual p values following ANOVA. All tests were two-sided, and p values < 0.05 were considered to indicate statistical significance. All experiments were repeated at least thrice.

## 3 Results

### 3.1 High expression of IKBKE correlates with high degree malignancy and poor prognosis in glioma patients

**Figure 1:**
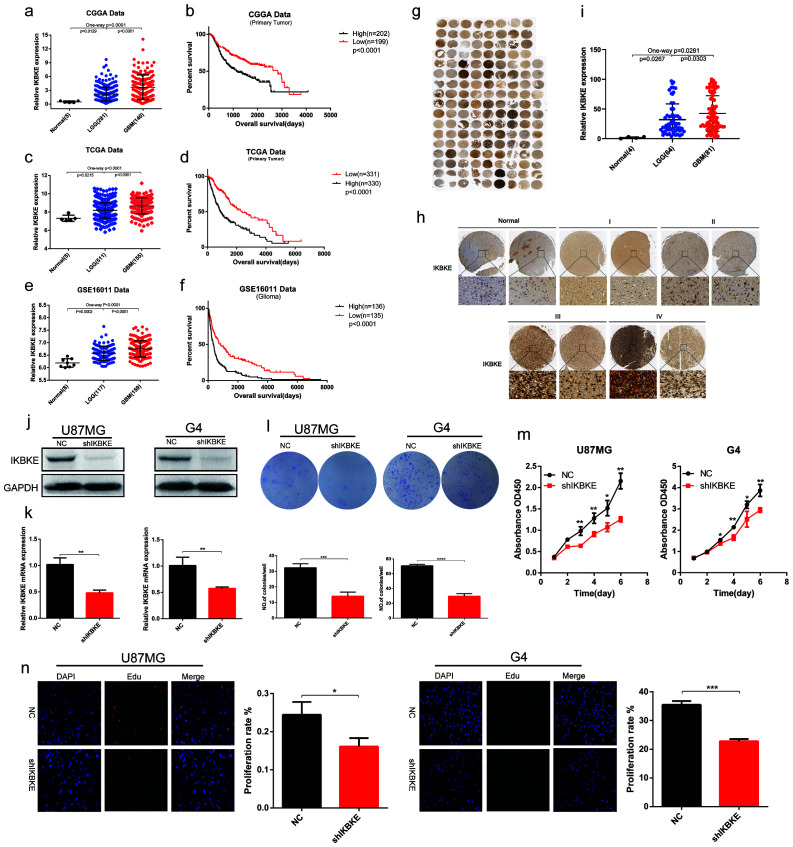
The expression of IKBKE is related to glioma grade and prognosis. Silencing IKBKE inhibits glioma cell proliferation. The levels of IKBKE were analyzed in non-tumor brain and glioma tissues of different grades **(a, c, e)** in the CGGA,TCGA and GSE16011 cohort. A Kaplan-Meier survival curve was used to examine the relationship between expression of IKBKE and the survival of patients with low grade gliomas and high grade gliomas **(b, d, f)** in the CGGA,TCGA and GSE16011 cohort. The IKBKE protein images in the entire tissue microarrays **(g)**. Representative images of IKBKE expressed on nontumor and glioma tissues of different grades **(h)**. The IKBKE protein expression is positively correlated with glioma grade in the entire tissue microarrays **(i)**. Western blotting and qPCR represent IKBKE expression in U87 and G4 cells infected with IKBKE sh-RNA **(j, k)**. Total cell proliferation ability was assessed by colony formation **(l)**, CCK8 assays **(m)** and EdU assays **(n)** in U87 and G4 cells. The asterisks denote significance (*p < 0.05, **p < 0.01, and ***p < 0.005).

To explore the function of IKBKE in glioma, we first analysed its expression in glioma and non-tumour brain tissues using the CGGA database, which includes five non-tumour brain and 421 primary glioma samples. The 421 glioma samples comprised 140 WHO II, 141 WHO III, and 140 WHO IV samples. IKBKE was found to be significantly overexpressed in GBM (WHO IV) compared to low-grade gliomas (LGGs), which include WHO I, WHO II, and WHO III (**Figure 1a**). Moreover, further analysis showed that the expression levels of IKBKE were significantly increased in glioma samples compared to those in normal brain samples (**Figure 1a**). We used Kaplan–Meier survival curves to analyse the correlation between IKBKE expression and the overall survival (OS) of patients with glioma. The results shown that glioma patients with higher IKBKE levels had poor overall survival (**Figure 1b**).

We then performed the same analysis in the TCGA and GSE16011 databases. These results were consistent with the previous analysis of the CGGA database (**Figures 1c–f**). To further investigate the clinical relevance of IKBKE in gliomas, we performed immunohistochemical staining with an antibody against IKBKE on glioma tissue microarrays (TMAs) consisting of 145 glioma samples and 4 normal brain samples. The results showed that IKBKE expression was higher in GBMs than in LGGs (**Figures 1g–i**). Furthermore, IKBKE expression was lower in normal brain samples than in glioma samples. These results indicate that IKBKE expression is positively correlated with glioma malignancy and negatively correlated with OS in patients with gliomas.

### 3.2 Knock-down of IKBKE inhibits GBM cells proliferation

IKBKE plays a key role in GBM progression (12, 18). To further explore the biological function of IKBKE in GBM cells, we constructed IKBKE-downregulated GBM cells using short hairpin RNA (shRNA). We used western blotting and qRT-PCR to detect the downregulation effect and found that the high expression level of endogenous IKBKE was suppressed in GBM U87MG cells and G4 cells (**Figures 1j, k**). As shown in **Figure 1l**, the U87MG and G4 cells with IKBKE downregulation significantly decreased the colony formation ability of cell colony formation. Moreover, CCK-8 and EdU assays showed that the proliferation of U87MG shIKBKE and G4 shIKBKE cells decreased markedly (**Figures 1m, n**). In summary, these results indicate that the inhibition of IKBKE expression significantly inhibited the growth of GBM cells.

### 3.3 IKBKE directly phosphorylates Amotl2 at s166

**Figure 2:**
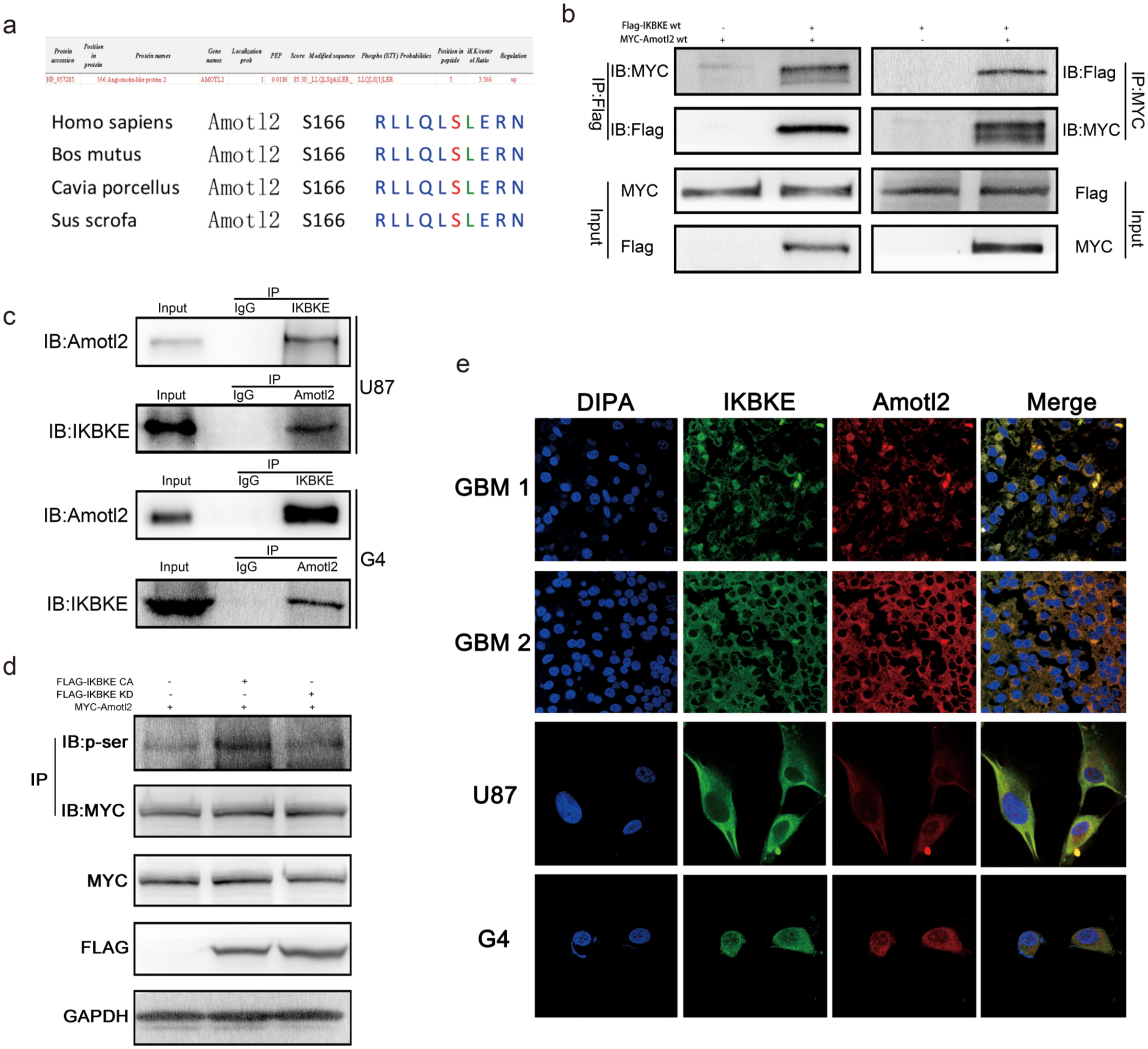
IKBKE directly phosphorylates Amotl2 at s166. The mass spectrometry (MS) analysis of U87MG cells transfected with shIKBKE or shNC lentivirus **(a)**; 293T cells were transfected with Flag-IKBKE and MYC-Amotl2 plasmids, immunoprecipitated with anti-Flag or anti-MYC antibody and immunoblotted with anti-MYC or anti-Flag antibody **(b)**. For endogenous IKBKE and Amotl2 interaction, U87MG and G4 cells were immunoprecipitated with anti-IKBKE or anti-Amotl2 and detected with anti-Amotl2 or anti-IKBKE antibody **(c)**. HEK293 cells were transfected with the indicated plasmids. Cells were immunoprecipitated with anti-Myc antibody and immunoblotted with indicated antibodies **(d)**; Immunoflourescence showing the protein distribution of IKBEK and Amotl2 in glioma cells and glioma tissues **(e)**.

Protein kinases (PKs) are enzymes that catalyse protein phosphorylation (9). Protein phosphorylation is an important component of many signal transduction pathways, and the most important cellular processes involve protein phosphorylation (34). To investigate the molecular mechanisms for IKBKE in glioblastoma, we performed protein mass spectrometry (MS) analysis of U87MG cells transfected with shIKBKE or shNC lentivirus. Among the mass proteins, we found that Amotl2 was highly phosphorylated at s166 in U87MG, and the amino acid sequence surrounding s166 was highly conserved in Amotl2 among multiple species (**Figure 2a**). To determine whether IKBKE binds and phosphorylates Amotl2. First, we transfected IKBKE-wt and Amotl2-wt plasmids into 293T cells, and immunoprecipitation analysis indicated that exogenous IKBKE and Amotl2 could interact directly (**Figure 2b**). In addition, we found that IKBKE and Amotl2 interacted with each other in U87MG and G4 cells (**Figure 2c**). These results indicated that IKBKE complexed with Amotl2 in GBM cells.

We next investigated whether IKBKE phosphorylated Amotl2 using an *in vivo* kinase assay. We co-transfected MYC-Amotl2 wt and either Flag-IKBKE CA, Flag-IKBKE dominant negative (DN), or vector into 293T cells. After incubation for 24 h, we used an anti-MYC antibody to immunoprecipitate Amotl2, which was then separated by SDS-PAGE. The results showed that p-Ser was higher in co-transfected MYC-Amotl2 and Flag-IKBKE CA than in the other groups (**Figure 2d**). These data indicated that IKBKE can phosphorylate Amotl2 at s166. In addition, we performed immunofluorescence staining on GBM samples and GBM cells and found that IKBKE colocalised with Amotl2 in the cytoplasm (**Figure 2e**). This further illustrated the possibility of an interaction between IKBKE and Amotl2.

### 3.4 IKBKE regulates the stability of Amotl2 through the ubiquitin–proteasome pathway

**Figure 3:**
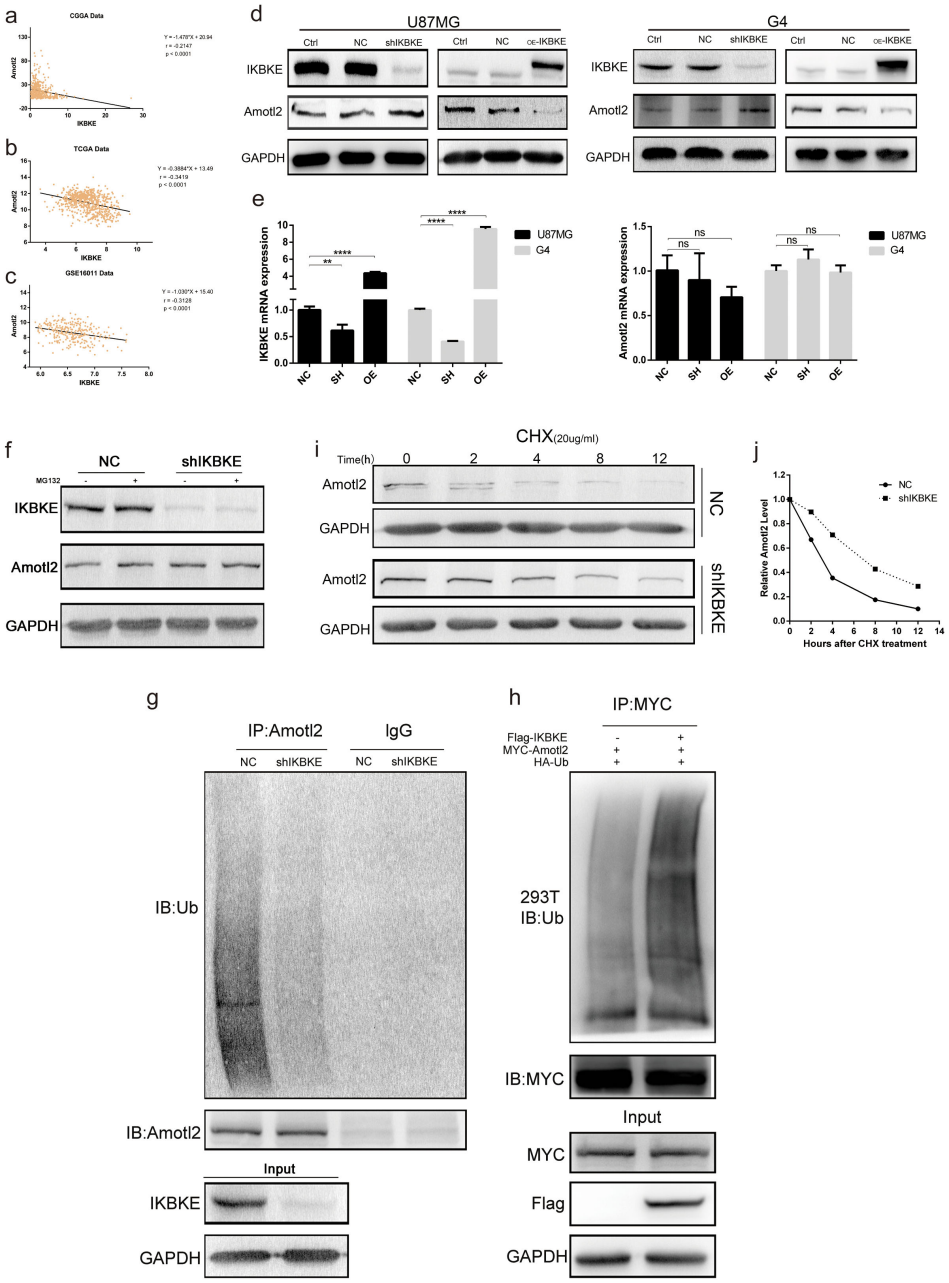
IKBKE regulates the stability of Amotl2. Correlation analysis of IKBKE mRNA and Amotl2 mRNA in the CGGA, TCGA and GSE16011 cohort **(a–c)**.The Amotl2 protein and mRNA level in glioma cells transfected with IKBKE-shRNA or IKBKE-overexpression lentivirus **(d, e)**. Amotl2 expression in IKBKE-knockdown U87 cells and control cells was measured after treatment with or without 20 μM MG132 for 12 h **(f)**. Measurement of Amotl2 in cell lysates harvested at 0, 2, 4, 8 and 12 h after the addition of CHX (100 μM) to arrest protein synthesis **(i)**. IKBEK promotes ubiquitination degradation of amotl2 **(g, h)**. The asterisks denote significance (*p < 0.05, **p < 0.01, ***p < 0.005, and ****p< 0.0001).

The CGGA database was used to perform a correlation analysis based on IKBKE and Amotl2 expression. The results indicated a negative correlation between the expression of IKBKE and Amotl2, and a high expression of IKBKE correlated with a low expression of Amotl2 in glioma samples (**Figure 3a**). In addition, we found that the correlations between IKBKE and Amotl2 in the TCGA and GSE16011 databases were consistent with the previous analysis in the CGGA database (**Figures 3b, c**).

To investigate the molecular mechanism of IKBKE regulation of Amotl2, U87MG and primary GBM cell line G4 were selected to construct stable knockdown and overexpression cell lines using lentivirus, respectively (**Figures 3d, e**). We detected the Amotl2 protein and mRNA levels after knockdown or overexpression of IKBKE in U87MG and G4 cells. The results showed that shIKBKE increased the protein expression of Amotl2, while oe-IKBKE downregulated the protein expression of Amotl2 (**Figure 3d**). However, after IKBKE knockdown or overexpression, the mRNA level of Amotl2 was not significantly different from that in the NC group (**Figure 3e**). These data indicated that IKBKE may regulate Amotl2 through a post-transcriptional pathway.

After treatment with the proteasome inhibitor MG132, we observed that the IKBKE-induced reduction in Amtol2 expression was blocked (**Figure 3f**). Next, we analysed Amtol2 ubiquitination levels in IKBKE-knockdown U87 cells. IKBKE knockdown decreases Amtol2 ubiquitination, indicating that IKBKE may regulate Amtol2 stability by promoting polyubiquitin degradation (**Figure 3g**). We also performed *in vivo* ubiquitination assays and observed that the active form—but not the DN form—of IKBKE promoted Amtol2 ubiquitination (**Figure 3h**). To measure the kinetics of Amtol2 degradation, cycloheximide was used to inhibit protein synthesis. The Amtol2 protein degraded faster in the control cells than in the shIKBKE cells (**Figures 3i, j**). These results implied that IKBKE inhibition promotes Amotl2 stability by decreasing its ubiquitination and degradation.

### 3.5 IKBKE regulates YAP1 expression and localization through Amotl2

**Figure 4:**
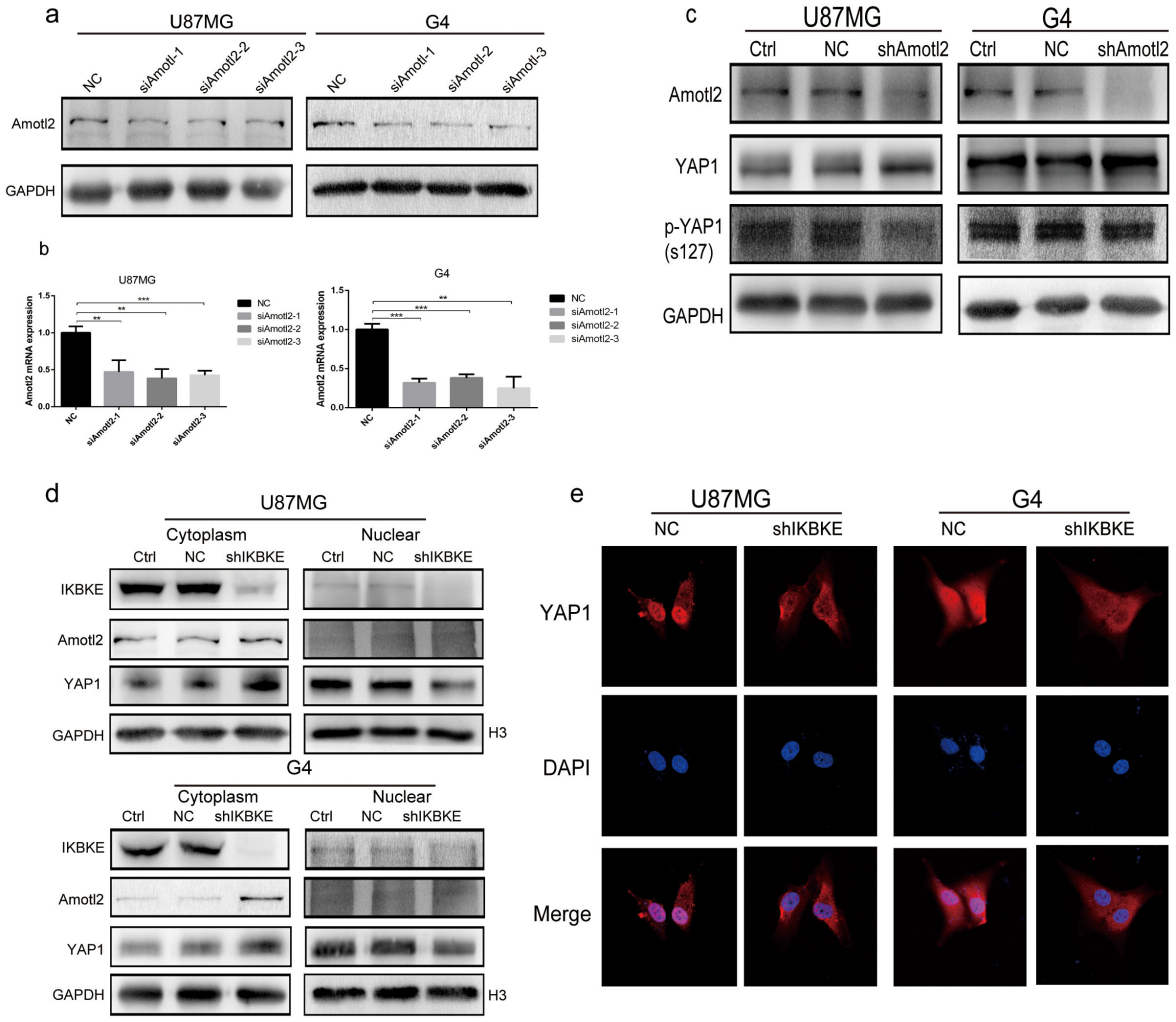
IKBKE regulates YAP1 expression and localization through Amotl2. Western blotting and qPCR represent Amotl2 expression in U87 and G4 cells infected with Amotl2 si-RNAs **(a, b)**. U87 and G4 cells were transfected with Amotl2-shRNA, and the expressions of YAP1 and P-YAP1(s127) were detected by western blot **(c)**. Expression of YAP1 in the cytoplasmic and nuclear fractions was examined by western blot and immunofluorescence **(d, e)**. The asterisks denote significance (*p < 0.05, **p < 0.01, and ***p < 0.005).

Amotl2 was found to be a potent inhibitor of the Hippo pathway effector YAP1, and Amotl2 can sequester YAP1 in the cytoplasm via protein–protein interactions or in a LATS-dependent manner (23, 28, 35, 36). Knockdown of Amotl2 in Madin–Darby canine kidney cells leads to YAP1 activation, which induces YAP1 target gene expression, attenuates YAP1 phosphorylation, and promotes the accumulation of YAP1 in the nucleus (24). Therefore, we hypothesised that IKBKE regulates YAP1 expression by regulating Amotl2 expression. First, we used qPCR and western blotting to detect the mRNA and protein levels of Amotl2 after U87MG and G4 cells were transfected with three siRNAs (**Figures 4a, b**). The results showed that siRNA#2 had a higher efficiency and was thus used in the subsequent analysis. We then detected YAP1 protein expression after Amotl2 knockdown, and the results showed that reduced Amotl2 could markedly stabilise the YAP1 protein (**Figure 4c**). Next, we demonstrated that IKBKE knockdown in U87 and G4 cells dramatically elevated the Amotl2 protein and suppressed the YAP1 protein and downstream targets of the Hippo pathway (**Supplementary Figure 2**). In contrast, the overexpression of IKBKE in U87 and G4 cells decreased Amotl2 protein expression and increased YAP1 protein expression (**Supplementary Figure 2**).

Subcellular fractionation demonstrated that the downregulation of IKBKE stabilised Amtol2, which triggered a reduction in YAP1 in the nuclear protein extracts of U87 and G4 cells, but not in the cytoplasm (**Figure 4d**). We provided western blot results of nuclear/cytoplasmic fractionation protein samples, which quantitatively measured the protein abundance of nuclear YAP1 and cytoplasmic YAP1 in NC and shIKBKE groups. The densitometry quantification of fractionated blots clearly confirmed that IKBKE knockdown inhibits nuclear translocation of YAP1, which is consistent with the qualitative IF observation. In addition, immunofluorescence analysis further confirmed the change in cytoplasmic and nuclear localisation of YAP1 when IKBKE was silenced (**Figure 4e**). The immunofluorescence phenotypes were reproduced in two distinct glioma cell lines (U87MG and primary G4 glioma cells), eliminating the possibility of cell-line-specific accidental bias. All IF images were randomly captured without manual selection of preferred fields, and the representative pictures shown in the figure accurately reflect the universal phenotype observed across dozens of visual fields during the original experiment.

**Figure 5:**
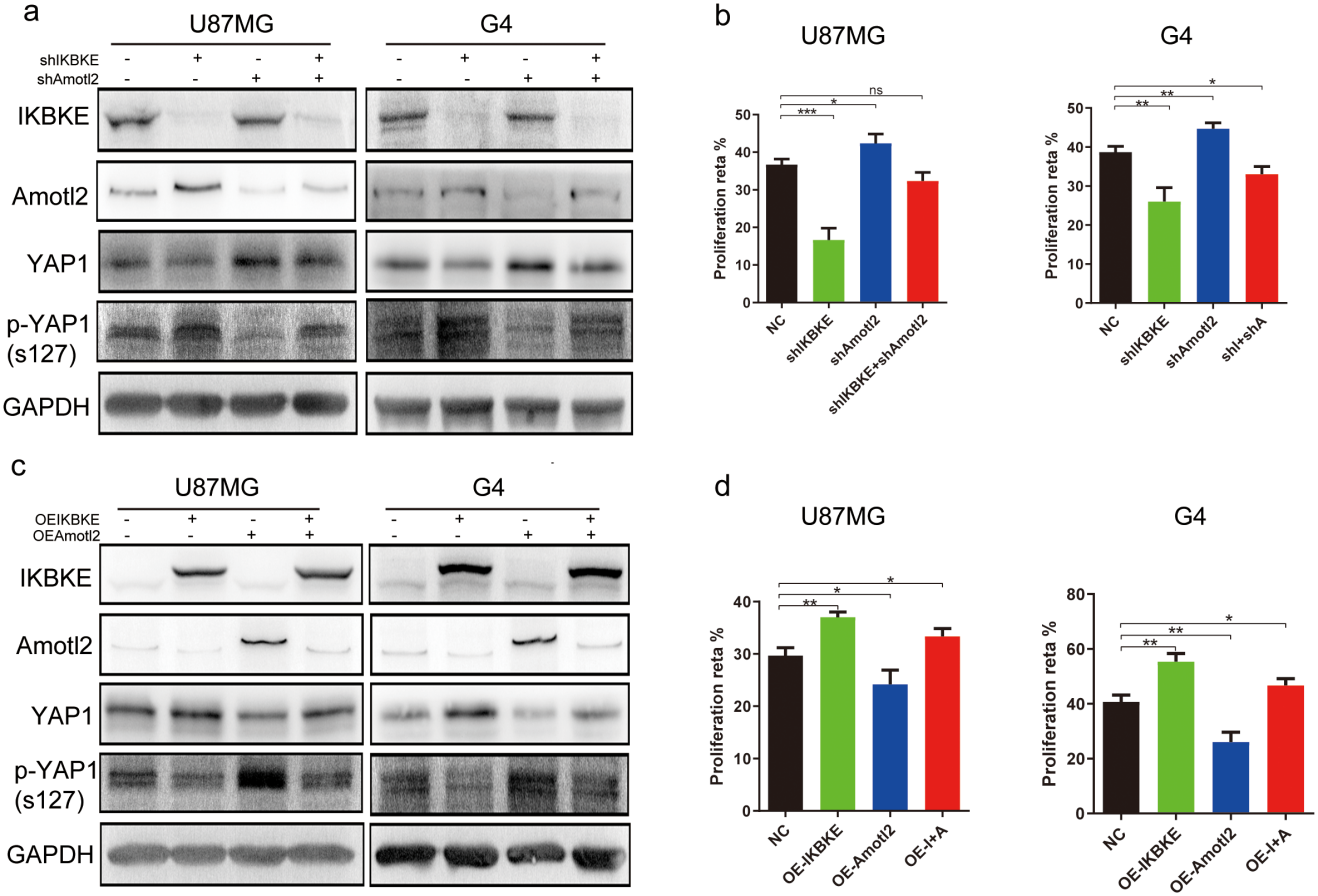
IKBKE regulate the expression of YAP1 through Amotl2. IKBKE, Amotl2, YAP1 and P-YAP1(S127) protein levels in glioma cells transfected with IKBKE-shRNA and Amotl2- shRNA, or transfected with IKBKE--overexpression lentivirus and Amotl2-overexpression lentivirus were detected by immunoblotting **(a, c)**. Total cell proliferation ability was assessed by EdU assays in glioma cells transfected with IKBKE-shRNA and Amotl2-shRNA, or transfected with IKBKE--overexpression lentivirus and Amotl2-overexpression lentivirus **(b, d)**. The asterisks denote significance (*p < 0.05, **p < 0.01, and ***p < 0.005).

To clarify that IKBKE can regulate the expression of YAP1 through Amotl2, we first knocked down IKBKE and Amotl2. The results showed that if Amotl2 was knocked down after IKBKE silencing, a part of the effect of YAP1 reduction caused by IKBKE silencing could be reversed (**Figure 5a**). The EdU assay results showed that if Amotl2 was knocked down after IKBKE, the cell proliferation induced by IKBKE silencing could be reversed (**Supplementary Figure 3a**).

Furthermore, we performed a recovery experiment to clarify the relationship among IKBKE, Amotl2, and YAP1. In GBM cells overexpressing IKBKE, Amotl2 overexpression partially reversed the IKBKE-induced increase in YAP1 expression (**Figure 5c**). The EdU assay showed that the overexpression of IKBKE group had the strongest proliferation ability, while the overexpression of IKBKE followed by the overexpression of Amotl2 cells significantly inhibited the proliferation ability (**Supplementary Figure 3b**).

### 3.6 IKBKE downregulation suppresses tumour growth in xenograft models

**Figure 6:**
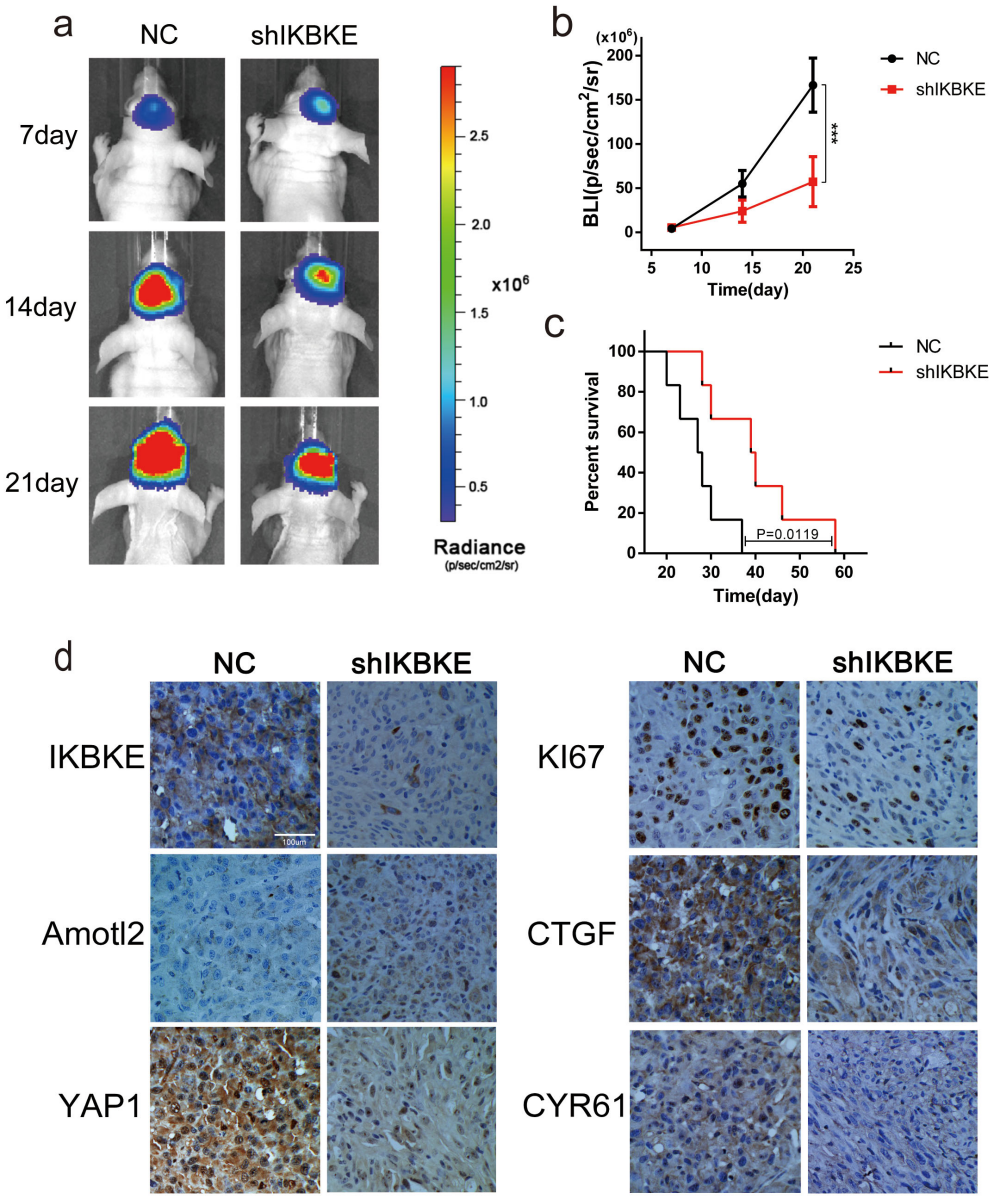
IKBKE regulates glioma growth *in vivo*. Bioluminescent images **(a)** and bioluminescent intensity **(b)** of mice on days 7, 14, and 21 after implantation. Survival rate of the glioma-bearing mice **(c)**. Representative images of IHC staining for IKBKE, Amotl2, YAP1, KI67, CTGF and CYR61 in tumors **(d)** (× 200 magnifcation). The asterisks denote significance (*p < 0.05, **p < 0.01, and ***p < 0.005).

U87 cells transfected with lentiviruses (sh-Ctrl or shIKBKE) expressing luciferase were stereotaxically injected into the brains of the nude mice. *In vivo* bioluminescence imaging analysis revealed that tumour growth was significantly inhibited in the shIKBKE group compared to that in the control group (**Figures 6a, b**). Likewise, the shIKBKE group was associated with a longer survival rate (**Figure 6c**). The expression levels of IKBKE, Amtol2, and Ki-67 were evaluated using immunohistochemistry (**Figure 6d**). Compared to the control group, the expression of Amtol2 increased, whereas the YAP1, CTGF, and Cyr61 expression levels simultaneously decreased in the tumour tissues of the IKBKE-silenced group, which was consistent with the *in vitro* results. Ki-67 is a marker of cell proliferation and predicts tumour progression and recurrence. The number of Ki67-positive cells was reduced in glioma sections derived from IKBKE shRNA compared to that in cells transfected with control shRNA. Taken together, these findings demonstrate that IKBKE knockdown inhibits the *in vivo* tumourigenicity of glioma cells.

## 4 Discussion

IKBKE has been implicated as an oncogenic protein that is upregulated and activated in various cancers (8). IKBKE overexpression could result in malignant transformation in multiple cell types, and IKBKE overexpression is significantly correlated with poor survival in patients with GBM (12, 16, 18, 19). Thus, IKBKE overexpression plays an important role in glioma and is a promising therapeutic target. We have conducted Kaplan-Meier survival analysis using CGGA, TCGA and GSE16011 glioma cohort data, which demonstrated that elevated IKBKE predicts poor overall survival of glioma patients.

One of the possible main functions of the Amot family is to integrate signalling pathways to regulate cellular shape (37–39). Of particular interest are the recent findings that Amot proteins are involved in controlling the Hippo pathway. Amot proteins associate with the Hippo effector YAP1 and negatively regulate its activity by sequestration in the cytoplasm (25, 27, 35). Here, we demonstrated that IKBKE expression is negatively correlated with the abundance of Amotl2 in GBM cell lines as well as glioma tissues, suggesting that IKBKE may also function through the regulation of Amtol2. The overexpression and downregulation of IKBKE affected the levels of Amtol2 but did not alter the mRNA levels in GBM cells. These results indicate that IKBKE regulates Amtol2 at the post-translational level. To elucidate the possible mechanism, we demonstrated that IKBKE directly binds to and phosphorylates Amtol2 at s166. Furthermore, our analysis showed that IKBKE downregulation stabilised Amtol2 by reducing ubiquitination. Moreover, the IKBKE-mediated phosphorylation of Amtol2 (Ser166) can trigger YAP1 nuclear–cytoplasmic translocation and reduce the ubiquitination and degradation of YAP1. At present, we do not have purified active IKKα/IKKβ/TBK1 proteins available to complete this *in vitro* kinase assay. However, we have added this question as a key future research direction and explained the potential biological meaning of testing their activity toward Amotl2 S166 for follow-up study.

Here, we investigated the function of IKBKE in an intracranial mouse model *in vivo*. The growth of tumours derived from the IKBKE knockdown group was significantly decreased compared to that in the control group. We hypothesised that impairments in GBM cell proliferation and growth that alleviate neoplastic extrusion towards the normal brain are responsible for different survival rates. The downregulation of IKBKE in GBM lesions is linked to significantly increased Amtol2 expression and decreased YAP1 expression, confirming the relationship between IKBKE and Hippo by regulating Amtol2 *in vivo*. Our integrated genetic and biochemical data adequately demonstrate that Amotl2 undergoes proteasome-mediated degradation upon IKBKE activation. Multiple independent peer-reviewed studies have validated endogenous Amotl2-YAP1 physical interaction in glioblastoma models via co-immunoprecipitation assays (1). The fundamental binding between Amot family proteins and YAP was originally identified and biochemically verified in multiple cell systems, including endogenous co-IP evidence (2). Mechanistically, phosphorylation of Amotl2 by upstream kinases disrupts this Amotl2-YAP complex to drive YAP nuclear translocation, consistent with our IKBKE-mediated regulatory model.

**Figure 7:**
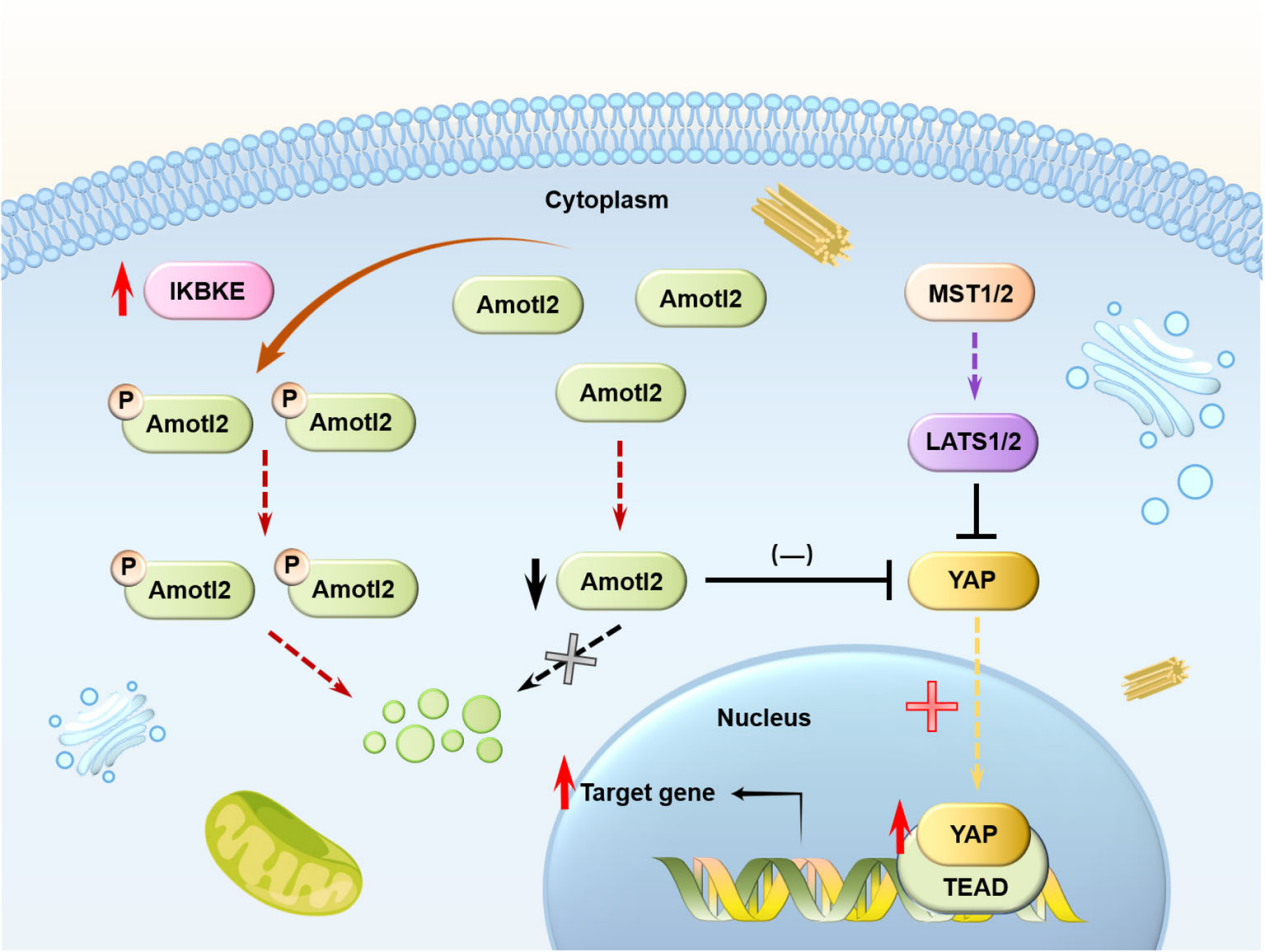
Diagram representing a proposed model of IKBKE regulation of Amotl2 leading to glioblastoma growth.

Accumulating substrates of IKBKE have recently been identified, all of which contribute to inflammation (40, 41) and tumourigenesis (8, 9, 42). Among these, LATS (12), NF-kb (10), AKT (17, 43), IRF3 (15), and STAT (13, 19)have been reported to control GBM growth. However, the potential functions and underlying mechanisms of IKBKE have not yet been defined. Mechanistically, our data demonstrate that IKBKE can phosphorylate Ser166 of Amtol2, resulting in the blockade of its YAP-binding properties, leading to the activation of YAP target genes (**Figure 7**).

## 5 Conclusions

Our study reveals a crucial role of IKBKE in accelerating GBM growth and highlights a potential therapeutic target to combat GBM by targeting IKBKE kinase. Discovering whether these results are generalizable to the treatment of other cancers will be interesting.

## Data Availability

The raw data supporting the conclusions of this article will be made available by the authors, without undue reservation.

## Supplementary material

Supplementary Figure 1 Total Amotl2 western blot gray scale analyze. **Figure 3d**. Amotl2 protein levels (Western blot grayscale analysis) **(a,b,c,d)**; **Figure 3f**. Amotl2 protein levels (Western blot grayscale analysis) **(e)**; **Figure 4a**. Amotl2 protein levels (Western blot grayscale analysis) **(f,g)**; **Figure 4d**. Amotl2 protein levels (Western blot grayscale analysis) **(h,i)**; **Figure 5a, c**. Amotl2 protein levels (Western blot grayscale analysis) **(j,k,I,m)**. (*p < 0.05, **p < 0.01, and ***p < 0.005).

Supplementary Figure 2 Amotl2, YAP1, P-YAP1(S172), CTGF and Cyr61 protein levels in U87 and G4 cells transfected with IKBKE-shRNA or IKBKE-overexpression lentivirus were detected by immunoblotting.

Supplementary Figure 3 Total cell proliferation ability was assessed by EdU assays in glioma cells transfected with IKBKE-shRNA and Amotl2-shRNA, or transfected with IKBKE--overexpression lentivirus and Amotl2-overexpression lentivirus.
